# Case Report: Prenatal clues and postnatal evolution: a case of williams syndrome diagnosed following progressive cardiovascular phenotypes

**DOI:** 10.3389/fped.2026.1831676

**Published:** 2026-07-09

**Authors:** Yuzhou Zeng, Yu Huang, Shilin Zeng, Wanying Yang, Qiutong Li, Jingrong Liu, Danping Huang, Hongying Wang

**Affiliations:** Department of Ultrasound, Guangzhou Medical University Guangzhou Women and Children’s Medical Center, Guangzhou, China

**Keywords:** cardiovascular phenotype evolution, chromosomal microarray analysis, coarctation of the aorta, fetal growth restriction, prenatal ultrasound, Williams syndrome

## Abstract

**Background:**

Williams syndrome (WS) is a rare genetic disorder often missed prenatally due to nonspecific presentations.

**Case presentation:**

We describe an infant with Williams syndrome diagnosed after progressive cardiovascular changes during infancy. Prenatal ultrasonography showed fetal growth restriction, persistent left superior vena cava (PLSVC), left-right cardiac chamber disproportion (LRCCD), and suspected coarctation of the aorta (CoA). Early postnatal echocardiography revealed mild aortic isthmus hypoplasia without definite supravalvular aortic or pulmonary arterial stenosis. Serial follow-up subsequently demonstrated supravalvular aortic stenosis (SVAS) and right pulmonary artery stenosis by 7 months of age, and chromosomal microarray analysis (CMA) confirmed a 1.42 Mb deletion at 7q11.23. At 5 years and 3 months of age, the child remained clinically stable, with stable SVAS and near resolution of pulmonary arterial stenosis. A retrospective review of nine fetuses with prenatally confirmed 7q11.23 microdeletion showed that prenatal cardiovascular abnormalities were present in 6 cases, mainly involving the aortic and pulmonary arterial systems, often with extracardiac findings such as renal anomalies and fetal growth restriction (FGR).

**Conclusion:**

WS may show dynamic cardiovascular evolution from nonspecific prenatal or neonatal findings to characteristic vascular lesions during infancy. Fetal cardiovascular abnormalities combined with FGR or other extracardiac anomalies should raise suspicion for WS and prompt consideration of CMA. Serial postnatal echocardiography may help clarify prenatal clues and facilitate earlier diagnosis and management.

## 1 Introduction

Williams syndrome (WS) is a rare genetic disorder caused by a heterozygous microdeletion in the 7q11.23 chromosomal region. Clinical manifestations include cardiovascular anomalies, distinctive facial features, intellectual and behavioral disabilities, and infantile hypercalcemia (1). Early diagnosis of WS is crucial for initiating timely multi-system monitoring and intervention (2, 3). However, prenatal diagnosis poses significant challenges, with frequent missed diagnoses due to the lack of specific clinical presentations (4, 5). Fetal growth restriction (FGR) combined with congenital cardiovascular defects represents the most common intrauterine phenotype of WS (6, 7). Herein, we report a case of prenatally suspected coarctation of the aorta (CoA), disproportion between the left and right cardiac chambers and FGR on fetal ultrasonography. Postnatally, the neonate was confirmed to have hypoplasia of the aortic arch via echocardiography. Subsequent serial cardiac follow-up examinations revealed disease progression to supravalvular aortic stenosis (SVAS) and Pulmonary artery stenosis (PAS). The diagnosis of WS was ultimately confirmed by genetic testing at 7 months of age. This case highlights the inconsistency and progressive nature of cardiovascular phenotypes in WS between the prenatal and postnatal periods.

## 2 Case presentation

The patient was a G2P1 woman with an unremarkable medical history during pregnancy. She received routine prenatal care at an external hospital. First-trimester Down syndrome screening indicated a high risk of trisomy 18. Invasive prenatal diagnosis via chorionic villus sampling (CVS) was performed, and the results of fetal chromosomal karyotyping were normal. A fetal ultrasound examination at 25⁺ weeks of gestation revealed a persistent left superior vena cava (PLSVC), left-right cardiac chamber disproportion (LRCCD), and a suspected CoA.

**Figure 1:**
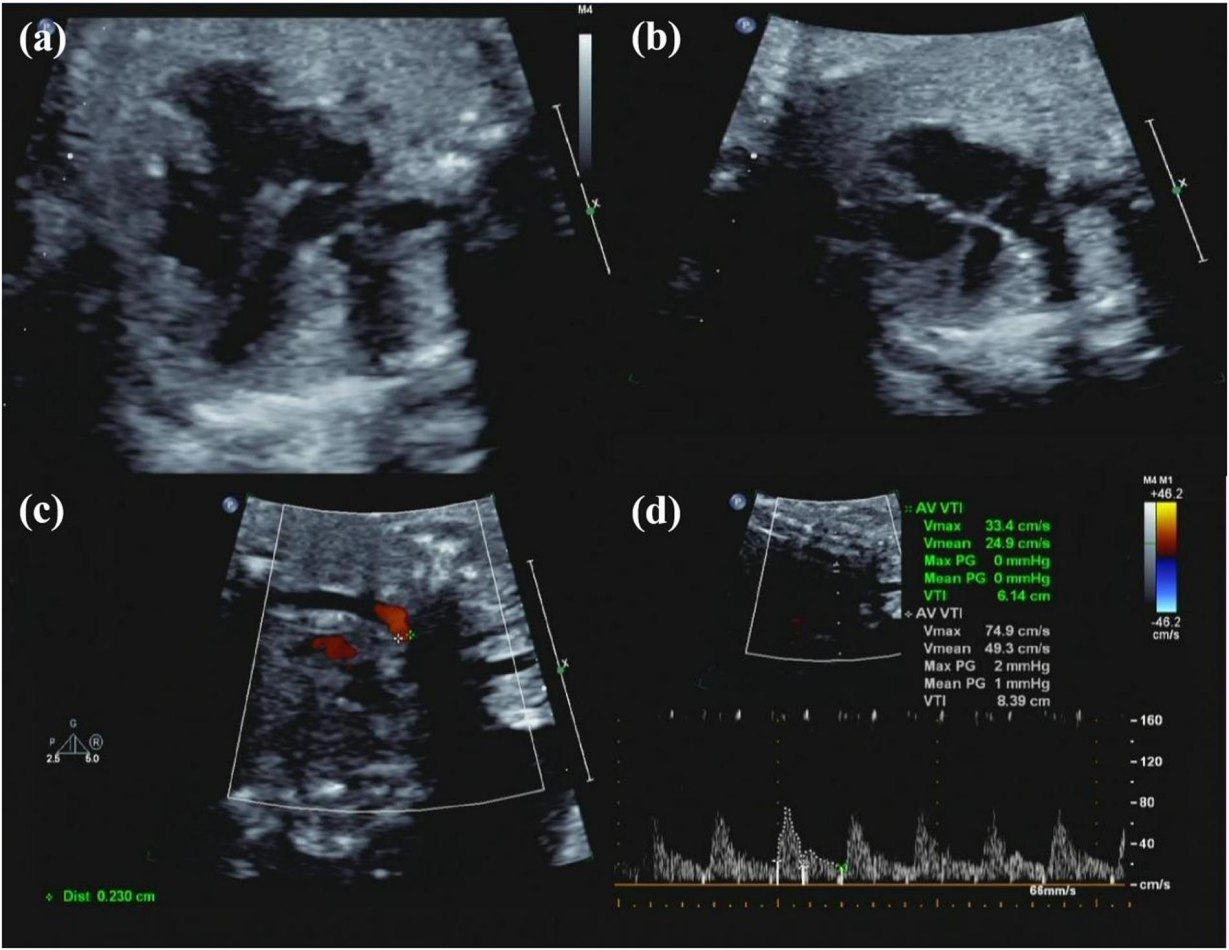
Prenatal fetal echocardiographic images. **(a)** No obvious SVAS was detected prenatally; **(b)** LRCCD was observed in the four-chamber view; **(c)** Mild narrowing of the aortic isthmus was noted; **(d)** accelerated flow with diastolic flow was observed in the aortic isthmus.

The patient was then referred to our institution for further evaluation. A repeat fetal echocardiogram at 33⁺ weeks of gestation confirmed the presence of fetal cardiovascular anomalies, including PLSVC, LRCCD, and a narrowed aortic isthmus with an internal diameter of 2.6 mm, which was suggestive of CoA (Figure 1). A third-trimester level III ultrasound scan at 36⁺ weeks of gestation showed the following fetal biometric parameters: biparietal diameter (BPD) 80 mm, head circumference (HC) 291 mm, abdominal circumference (AC) 262 mm, and femur length (FL) 58 mm. All these measurements were below the normal gestational age reference range [−2 standard deviations (SD), calculated based on a gestational age of 36⁺^3^ weeks], leading to a diagnosis of FGR.

Given the coexistence of fetal cardiovascular abnormalities and FGR, additional prenatal chromosomal microarray analysis (CMA) was recommended to improve the detection of submicroscopic chromosomal abnormalities. However, because these findings were recognized at a relatively advanced gestational age, the additional information provided by prenatal CMA was unlikely to substantially alter immediate pregnancy management. In addition, the patient had been referred from another region, making further testing less convenient. Together with the previously normal conventional karyotype, this reduced the family's willingness to pursue further invasive prenatal genetic testing. Therefore, after genetic counseling, prenatal CMA was not performed and the pregnancy was continued to delivery.

The neonate was delivered via cesarean section at 38⁺^1^ weeks of gestation, with a birth length of 41 cm and a birth weight of 1870g. The Apgar scores were within the normal range at all time points.

### 2.1 Neonatal period evaluation (0–28 days)

A transthoracic echocardiogram (TTE) was performed on the 3rd postnatal day. The key findings were as follows:

Aortic arch: The narrowest segment of the aortic isthmus measured approximately 2.7 mm in internal diameter, with maximum velocity (Vmax) = 1.8 m/s and pressure gradient (*Δ*P) = 12 mmHg. Post-stenotic dilation was noted in the distal segment.

Other cardiac structures: Persistent left superior vena cava (PLSVC) draining into the coronary sinus was confirmed. No significant SVAS or PAS was detected.

Diagnosis: Mild hypoplasia of the aortic isthmus; PLSVC.

### 2.2 Infantile period follow-up and diagnosis update (day 29 to 7 months of age)

The infant underwent regular follow-up at the pediatric cardiology specialist clinic. Physical examination findings: Blood pressure was 112/57 mmHg in both upper extremities and 131/57 mmHg in both lower extremities. Serial TTE re-examinations at 1, 4, and 7 months of age revealed progressive cardiovascular changes. The TTE at 1 month of age indicated unobstructed aortic blood flow with mildly accelerated flow in the bilateral pulmonary arteries. In contrast, the TTE studies at 4 and 7 months of age confirmed the development of SVAS and right pulmonary artery stenosis.

**Figure 2:**
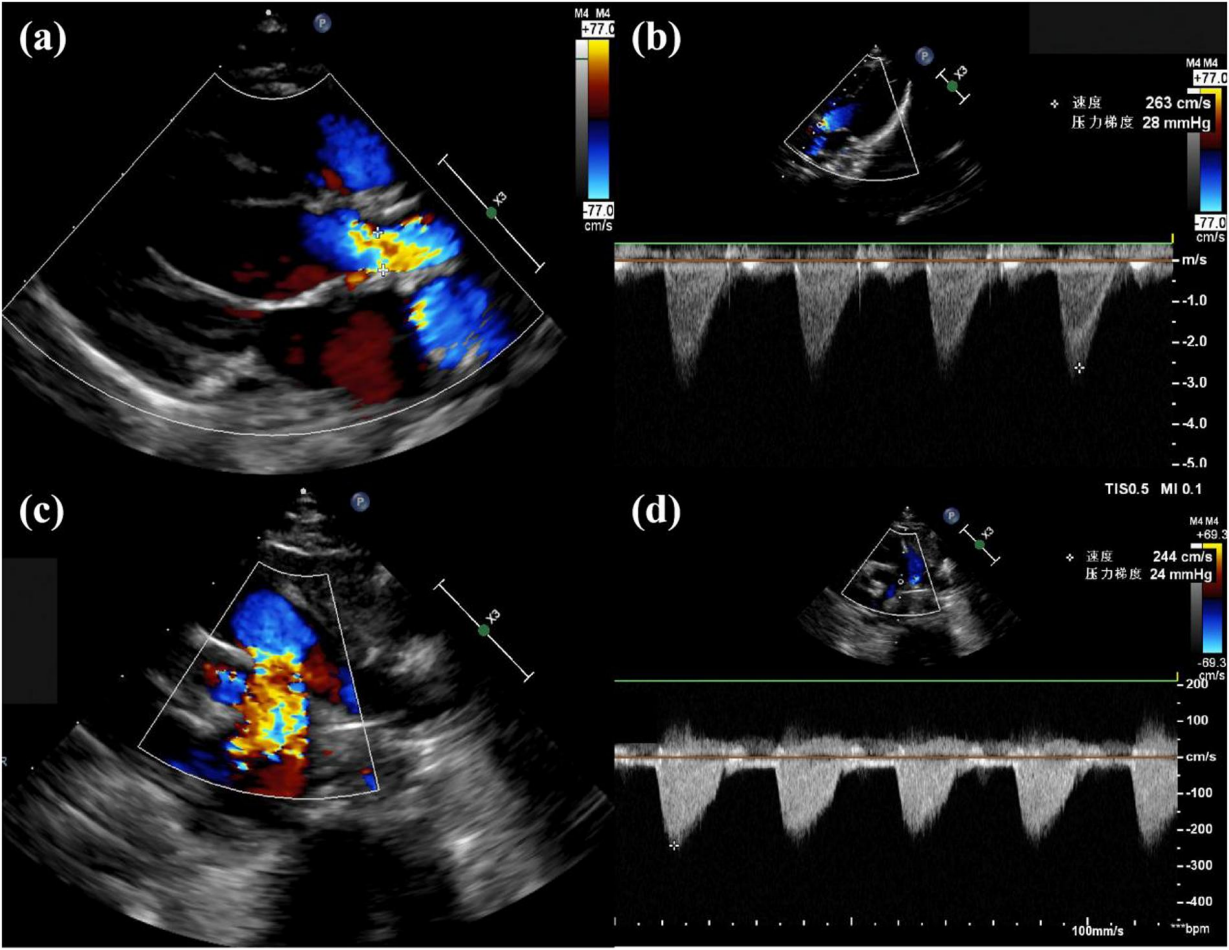
TTE findings of the infant at 7 months of age. **(a)** Color Doppler imaging showing aliased flow at the narrowed sinotubular junction; **(b)** Pulsed-wave Doppler demonstrating increased flow velocity at the sinotubular junction; **(c)** Color Doppler imaging revealing aliased flow at the focal stenosis of the right pulmonary artery ostium; **(d)** Pulsed-wave Doppler indicating elevated flow velocity in the right pulmonary artery.

Detailed TTE findings at 7 months of age were as follows (Figure 2):

SVAS: **moderate** narrowing of the sinotubular junction was noted, with the internal diameter reduced significantly to approximately 5.4 mm. The blood flow velocity in the ascending aorta was increased to 2.7 m/s, with a corresponding *Δ*P of 30 mmHg.

PAS: Focal stenosis was identified at the ostium of the right pulmonary artery, with an internal diameter of approximately 4.6 mm. The peak systolic flow velocity Vmax was 2.4 m/s, and the *Δ*P was 24 mmHg.

The infant’s cardiovascular phenotype underwent a distinct dynamic evolution: from an “isolated” CoA in the neonatal period, it rapidly progressed to the characteristic combined phenotype of SVAS (at the sinotubular junction) and PAS within the first 7 postnatal months (8). This progressive course may suggest that the underlying etiology is the typical elastin arteriopathy observed in Williams syndrome.

### 2.3 Definitive diagnosis

Given the infant’s characteristic facial features, including periorbital fullness and ptosis, dental abnormalities (small, widely spaced teeth), and cardiovascular manifestations, CMA was performed at 7 months of age. The results confirmed a 1.42 Mb microdeletion at the 7q11.23 locus, consistent with the diagnosis of WS. During hospitalization, serum calcium was 2.45 mmol/L, which was within the normal reference range. Electrocardiographic evaluation showed sinus rhythm with a normal PR interval (0.14 s), QT interval (0.288 s), and QTc (0.41), without significant ST-segment deviation or voltage abnormality. Three months later, repeat ECG showed sinus tachycardia, again without significant abnormalities in waveform morphology, intervals, or ST-T segments.

Following definitive diagnosis, systematic follow-up was initiated. Significant delays in neuromotor development were observed: independent sitting was achieved at 9 months, crawling at 10 months, assisted standing at 12 months, and independent walking at 15 months of age. Language development was also delayed, with the onset of intentional vocalizations occurring only after 9 months of age. Under the management of a multidisciplinary team (MDT) comprising specialists from pediatric cardiology, clinical genetics, developmental-behavioral pediatrics, and pediatric rehabilitation medicine, the infant received early developmental interventions and regular clinical monitoring.

**Figure 3:**
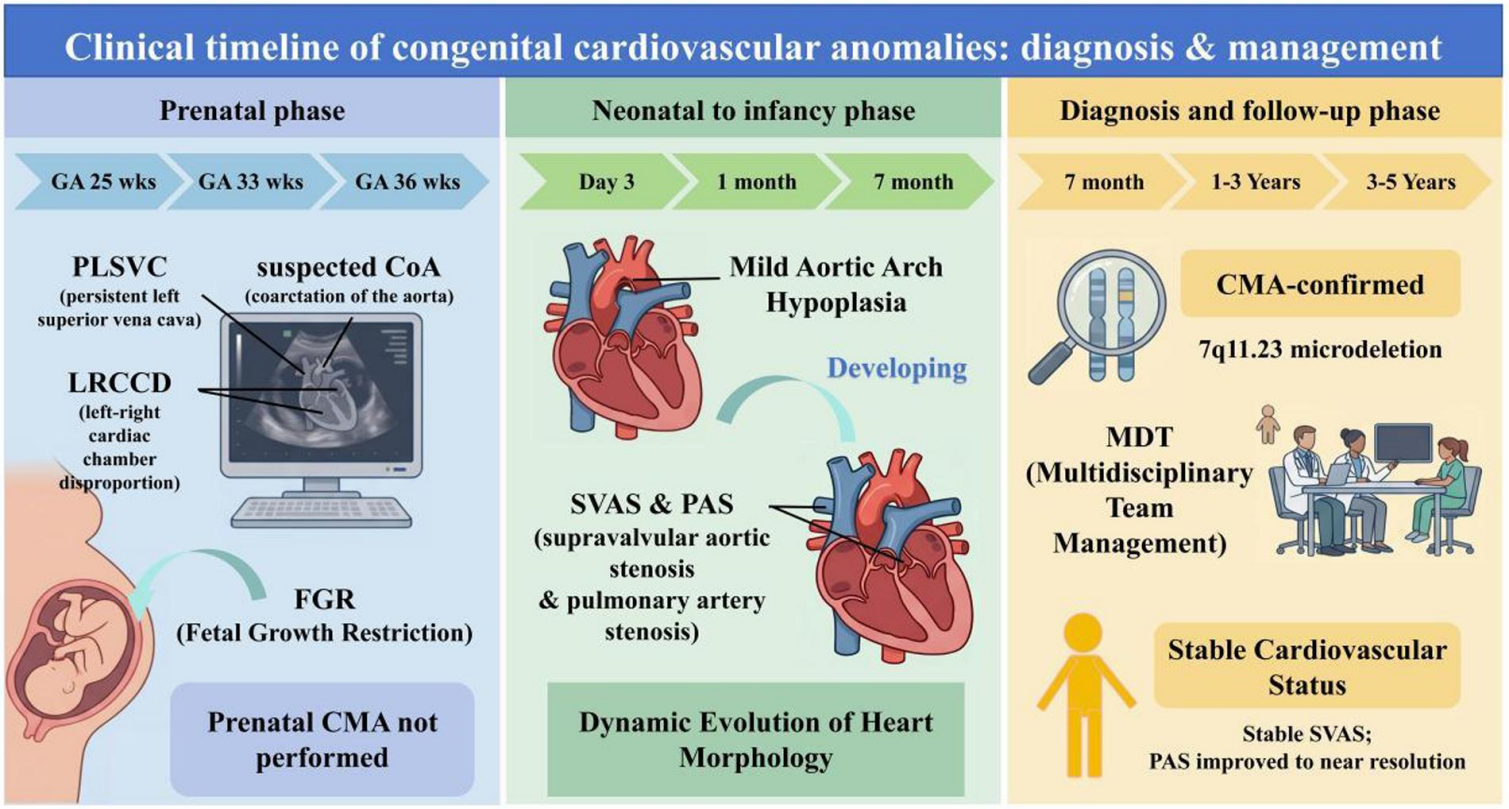
Timeline summarizing prenatal clues, postnatal cardiovascular progression, and diagnostic confirmation in the reported case of williams syndrome. Prenatal imaging showed PLSVC, LRCCD, suspected CoA, and FGR, whereas prenatal CMA was not performed. After birth, mild aortic arch hypoplasia evolved into SVAS and PAS by 7 months. CMA then confirmed a 7q11.23 microdeletion, and long-term MDT follow-up showed stable SVAS, near resolution of PAS, and no need for medical, surgical, or catheter-based treatment. GA, gestational age; PLSVC, persistent left superior vena cava; LRCCD, left-right cardiac chamber disproportion; CoA, coarctation of the aorta; FGR, fetal growth restriction; SVAS, supravalvular aortic stenosis; PAS, pulmonary artery stenosis; CMA, chromosomal microarray analysis; MDT, multidisciplinary team.

At the latest follow-up at 5 years and 3 months of age, the child remained in stable general condition. The cardiovascular lesions were managed conservatively with regular cardiology follow-up. During long-term follow-up, the PAS gradually improved to near resolution, whereas the SVAS remained stable. No medication, surgical intervention, or catheter-based intervention has been required to date. Steady progress was observed in physical growth, motor development, and cognitive abilities, which was attributed to the sustained rehabilitation training (Figure 3).

## 3 Discussion

This case illustrates the complete clinical trajectory of WS, from the identification of prenatal suggestive clues to the definitive diagnosis made during infancy. The core of this diagnostic process lies in the integrated recognition of its progressive phenotypic spectrum.The distinctive value of this case lies in the close longitudinal observation of postnatal phenotypic evolution, which in turn helped us retrospectively reinterpret the prenatal findings and identify fetal manifestations with potential diagnostic relevance.

First and foremost, the prenatal identification of “FGR complicated by specific cardiac malformations (suspected CoA)” constitutes an important warning sign, and a high index of suspicion for genetic syndromes including WS is warranted (9). This highlights the need to broaden the differential diagnosis and strengthen genetic counseling when fetal cardiac anomalies are accompanied by growth restriction or other multisystem abnormalities. At the same time, this case also illustrates the practical difficulty of prenatal recognition of WS. Although fetal cardiovascular abnormalities and FGR were present, these findings were not sufficiently specific to strongly suggest WS (10), especially in the absence of the more recognizable postnatal phenotype of supravalvular aortic stenosis and branch pulmonary artery stenosis. In addition, a normal conventional karyotype may reduce the perceived urgency for further genetic testing, while real-world factors such as geographic barriers and decision-making pressure in late gestation may further limit the uptake of prenatal CMA.

More importantly, the dynamic cardiovascular evolution in this case provided a key diagnostic clue. Prenatal and neonatal findings were relatively nonspecific and could easily be interpreted as an isolated congenital heart defect. However, the rapid development of SVAS and PAS during short-term follow-up is consistent with a classic manifestation of the characteristic elastin arteriopathy in WS (11). This evolution from relatively nonspecific early findings to a more characteristic multisite vascular phenotype served as the core diagnostic clue that strongly suggested WS and ultimately led to definitive genetic confirmation (12).

At the same time, the longer-term course of this patient showed that cardiovascular evolution in WS is not uniformly progressive. While the SVAS remained stable, the PAS gradually improved to near resolution. This observation is consistent with the heterogeneous natural history reported in the literature, in which systemic arterial lesions may persist or progress, whereas branch pulmonary artery stenosis may stabilize or regress (13).

To further delineate the prenatal ultrasound spectrum of WS, we reviewed nine fetuses diagnosed at our institution over the past decade. Their main prenatal features are summarised in Table 1. Cases were included if prenatal CMA confirmed a 7q11.23 microdeletion and corresponding prenatal ultrasound and/or fetal echocardiographic data were available for review. All 9 pregnancies were subsequently terminated for medical indications after prenatal diagnosis.

**Table 1:** Summary of prenatal ultrasonographic features in fetuses With 7q11.23 microdeletion.

Group	Case no.	Maternal age (years)	Gestational age (weeks)	Prenatal cardiovascular findings	Other prenatal ultrasonographic findings	Microarray result (size of 7q11.23 microdeletion)	Pregnancy outcome
Documented cardiovascular abnormalities	1	32	25	VSD; suspected CoA	Bilateral renal hypoplasia	1.42 Mb	TOP
	2	38	33	LRCCD; mild aortic hypoplasia; VSD	None	1.42 Mb	TOP
	3	27	34	LRCCD; mild aortic hypoplasia; accelerated flow with regurgitation in the RPA; tortuous DA	Cystic dilatation of the intra-abdominal UV	1.53 Mb	TOP
	4	32	35	SVAS; SVPS; bilateral PAS; mild aortic isthmus hypoplasia; tortuous DA; increased CTR; TR; small pericardial effusion; abnormal echogenicity at the RV apex, suggestive of NCCM	Bilateral renal enlargement with increased parenchymal echogenicity; bilateral renal cysts (suspected MCDK); left hydronephrosis; mild penile hypoplasia	1.25 Mb	TOP
	5	33	30	Mild stenosis of the pulmonary valve and branch pulmonary arteries	FGR	2.07 Mb	TOP
	6	34	22	pulmonary atresia; VSD; overriding aorta; accelerated flow velocity at the supravalvular aortic level; right-sided aortic arch	None	1.42 Mb	TOP
No documented cardiovascular abnormalities	7	23	30	None	FGR	1.44 Mb	TOP
	8	29	30	Fetal echocardiography not performed	Duodenal atresia	1.42 Mb	TOP
	9	31	23	None	Increased bilateral renal echogenicity; right MCDK	1.43 Mb	TOP

Data are presented as individual fetal cases with prenatally confirmed 7q11.23 microdeletion identified at our center over the past decade. The gestational age shown refers to the gestational age at which the principal abnormalities were first documented during prenatal ultrasound and/or fetal echocardiographic evaluation at our center. Because some cases were referred from other hospitals, this gestational age may not represent the earliest onset of abnormalities. All 9 pregnancies were subsequently terminated for medical indications after prenatal diagnosis. Cases were grouped according to whether prenatal cardiovascular abnormalities were documented by fetal echocardiography. Case 8 was included in the group without documented cardiovascular abnormalities because fetal echocardiography was not performed.

CoA, coarctation of the aorta; CTR, cardiothoracic ratio; DA, ductus arteriosus; FGR, fetal growth restriction; LRCCD, left-right cardiac chamber disproportion; MCDK, multicystic dysplastic kidney; NCCM, non-compaction cardiomyopathy; PAS, pulmonary artery stenosis; RPA, right pulmonary artery; RV, right ventricle; SVAS, supravalvular aortic stenosis; SVPS, supravalvular pulmonary stenosis; TOP, termination of pregnancy; TR, tricuspid regurgitation; UV, umbilical vein; VSD, ventricular septal defect.

Prenatal cardiovascular abnormalities were detected in 6 of the 9 fetuses (66.7%), predominantly involving the aortic and pulmonary arterial systems. Aortic lesions were observed in 5 cases, pulmonary arterial lesions in 4 cases, and ventricular septal defect (VSD) in 3 cases; a tortuous ductus arteriosus was noted in 2 cases. Extracardiac findings were also common, especially renal anomalies (4/9) and fetal growth restriction (2/9), supporting the concept that prenatal Williams syndrome may present as a multisystem disorder.

These findings are broadly consistent with previous prenatal studies (6, 7), which have reported recurrent aortic abnormalities, including coarctation-related lesions and SVAS, whereas PAS appears to be less frequently recognized prenatally. Because of the small sample size and heterogeneity in lesion classification across studies, direct comparisons should be made cautiously. Nonetheless, our data suggest that aortic lesions may be more readily detected than pulmonary arterial lesions during fetal assessment. Overall, these observations suggest that WS may present prenatally as a multisystem disorder in which cardiovascular abnormalities coexist with extracardiac findings.

## 4 Limitation

This study combined a single case with longitudinal prenatal and postnatal follow-up and a retrospective series focused on prenatal diagnoses. However, because the retrospective cases had no postnatal follow-up data, they were informative only for prenatal phenotypic characterization and not for assessing postnatal cardiovascular evolution.

## 5 Conclusion

This case highlights the dynamic cardiovascular evolution of WS, in which nonspecific prenatal or neonatal cardiovascular findings may progress to characteristic vascular lesions during infancy. When fetal cardiovascular abnormalities, particularly aortic arch abnormalities or pulmonary arterial lesions, coexist with FGR or other extracardiac anomalies, WS should be considered, and CMA may be warranted (14). In postnatally suspected cases, serial echocardiographic follow-up is important for identifying emerging vascular stenosis and for clarifying the clinical significance of earlier prenatal findings. Integrating prenatal ultrasonographic clues with postnatal cardiovascular evolution may facilitate earlier genetic diagnosis, timely multidisciplinary management, and individualized long-term follow-up.

## Data Availability

The original contributions presented in the study are included in the article/Supplementary Material, further inquiries can be directed to the corresponding author.
